# Impact of the COVID-19 Pandemic on Surgical Treatment Patterns for Colorectal Cancer in a Tertiary Medical Facility in Korea

**DOI:** 10.3390/cancers13092221

**Published:** 2021-05-06

**Authors:** Ju Yeon Choi, In Ja Park, Hyun Gu Lee, Eunhae Cho, Young Il Kim, Chan Wook Kim, Yong Sik Yoon, Seok-Byung Lim, Chang Sik Yu, Jin Cheon Kim

**Affiliations:** 1 Department of Surgery, Asan Medical Center, University of Ulsan College of Medicine, Seoul 05505, Korea; jychoi727@daum.net; 2 Department of Colon and Rectal Surgery, Asan Medical Center, University of Ulsan College of Medicine, Seoul 05505, Korea; gusrn1230@gmail.com (H.G.L.); cec1103@gmail.com (E.C.); illie8246@gmail.com (Y.I.K.); crscwkim@amc.seoul.kr (C.W.K.); yoonys@amc.seoul.kr (Y.S.Y.); sblim@amc.seoul.kr (S.-B.L.); csyu@amc.seoul.kr (C.S.Y.); jckim@amc.seoul.kr (J.C.K.)

**Keywords:** colorectal cancer, COVID-19, aggressiveness of cancer, minimally invasive surgery, pandemic

## Abstract

**Simple Summary:**

The COVID-19 pandemic is threatening to public health, including malignant disease. Fear of viral infection has influenced the diagnosis and treatment of colorectal cancer and may result in impairment of surgical and oncologic outcomes. Therefore, we need to analyze the influence of COVID-19 on surgical outcomes of colorectal cancer and provide guidance on proper diagnosis and treatment, including public messaging regarding appropriate healthcare.

**Abstract:**

Because of their reluctance to visit the hospital due to concerns about contracting coronavirus disease 2019 (COVID-19), patients with colorectal cancer have been affected by delays in care during the pandemic. This study assessed the effects of the pandemic on the clinical characteristics and surgical treatment patterns of colorectal cancer patients at a tertiary medical facility in Korea. Patients who underwent colorectal cancer surgery at our institution between March and September 2020 were analyzed. Clinicopathological and treatment characteristics were compared with those of patients who underwent surgery in 2018 and 2019. The patients who did not undergo tumor resection (4.1% vs. 1.8%, *p* < 0.001) and who received neoadjuvant treatment (16.7% vs. 14.7%, *p* = 0.039) were significantly higher during the COVID period. The minimally invasive approach was performed less during the COVID period (81.2% vs. 88%, *p* < 0.001). More patients in the COVID period required combined resection of organs adjacent to the tumor (4.8% vs. 2.8%, *p* = 0.017). Surgical aggressiveness, as shown by the proportion of patients undergoing minimally invasive surgery and adjacent organ resection, was significantly influenced by the pandemic. In addition, resectability decreased during the COVID period. These characteristics will likely influence long-term oncological outcomes, indicating the need for long-term monitoring of this cohort.

## 1. Introduction

The SARS-CoV-2 (severe acute respiratory syndrome coronavirus 2) virus, initially observed in Wuhan, China, and the cause of COVID-19 (coronavirus disease), has spread worldwide, with over 80 million people confirmed as having COVID-19 and over 1.7 million people dying from this disease. The World Health Organization (WHO) declared it a global pandemic, which is regarded as a public health emergency [1]. Continuation of the epidemic has altered the lifestyle of the population for over 1 year [2], including the requirement to wear masks and maintain social distance. The government of South Korea has recommended that patients visit hospitals only when experiencing severe symptoms [3]. People have minimized going outside their homes, even to hospitals, as recommended by disease control guidelines, to avoid contact with carriers of infection. Patients with cancer have not been excluded from these social restriction guidelines.

Due to the pandemic, the number of outpatients has decreased in South Korea [4]. In Italy, the number of patients diagnosed with cancer at secondary care facilities was 39% lower in 2020 than in 2018 and 2019, with the highest rates of decrease in patients diagnosed with colorectal (62%), bladder (66%), and prostate (75%) cancers [5]. Delays in diagnosis have raised concerns regarding avoidable cancer deaths. For example, the number of deaths due to colorectal (15.3–16.6%), breast (7.9–9.6%), lung (4.8–5.3%), and esophageal (5.8–6.0%) cancers during the COVID-19 lockdown increased markedly in the U.K. [6]. Although COVID-19 was found to have a negative impact on cancer patients, there have been no studies assessing the correlation between surgical outcomes and oncological aspects during the COVID-19 pandemic [7], suggesting that studies comparing outcomes before and during the COVID-19 pandemic may help guide treatment during the post-COVID-19 period [8,9].

Delays in cancer diagnosis lead to presentation at more advanced stages of disease and poorer clinical outcomes. Surgical interventions should not be limited to patients requiring urgent treatment, even during the COVID-19 pandemic [10]. Diagnostic delay may have an especially negative impact on patients with colorectal cancer [5,11]. Studies on surgical treatment patterns for colorectal cancer during epidemics are increasing. The present study therefore evaluated the treatment patterns and outcomes of colorectal cancer patients during the COVID-19 pandemic by comparing the demographic and clinical characteristics and surgical treatment patterns of colorectal patients treated at a tertiary medical facility in Korea before and during the pandemic.

## 2. Materials and Methods

This prospective study included patients with colon and rectal cancer who underwent surgery at the Asan Medical Center between March and September 2020. Since the first case of COVID-19 was detected in South Korea, the government announced measures to avoid the spread of infectious disease. The national health authorities of South Korea divided the COVID period into three stages: the early stage from 1 January to 17 February 2020, the group infection stage from 18 February to 5 May 2020, and the ongoing social distance stage beginning on 6 May 2020 [3]. In particular, awareness of SARS-CoV-2 was strong and personal hygiene was strictly implemented during the group infection and social distancing stages. All patients who underwent any surgical procedures for primary colorectal carcinoma from March to September 2020 were included. Patients with familial colorectal cancer, colorectal malignancies other than adenocarcinoma, and inflammatory bowel-disease-associated cancer were excluded, as were patients with recurrence or metastasis after primary cancer surgery. This research was approved by the Institutional Review Board (2017–1114).

Nasal swabs were taken from every patient in our institution for PCR (polymerase chain reaction) testing for coronavirus. In the absence of testing, surgical treatment was not permitted. Patients who had fever, sputum, cough, or any other respiratory symptoms underwent repeated COVID-19 testing. None of the patients included in the present study were positive for COVID-19.

Tumor characteristics and surgical outcomes were compared in patients who underwent surgery during 2020 (the COVID period) with those who underwent surgery during the same time periods in 2018 and 2019 (the pre-COVID period). Factors compared included demographic characteristics, tumor locations, resectability, use of a minimally invasive approach, TNM stage, resection of adjacent organs, use of neoadjuvant chemo- or radiotherapy, and postoperative major-surgery-related complications, which are defined as complications directly related to surgery for primary colorectal cancers requiring surgical or interventional management. All data were analyzed retrospectively. Categorical variables were compared using the chi-square test, and continuous variables were compared using t-tests. Statistical significance was assumed at a level of 5%. All statistical analyses were performed using IBM SPSS Statistics for Windows, version 21.0 (IBM Corp., Armonk, NY, USA).

## 3. Results

### 3.1. Demographic, Tumor, and Procedure Characteristics

**Figure 1:**
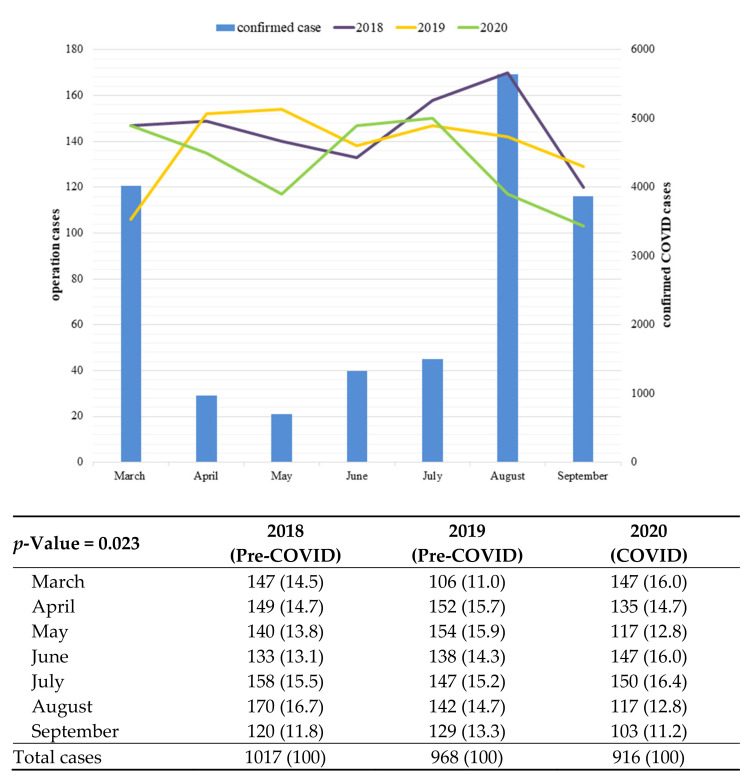
Monthly number of patients confirmed as having COVID-19 and undergoing colorectal cancer surgery from March through September 2018, 2019, and 2020.

The proportion of colorectal surgeries for primary colorectal cancer did not differ significantly during the COVID and pre-COVID periods (50.2% vs. 49.6%), but the number of colorectal cancer surgeries was 5.4% lower in 2020 than in 2019. The monthly proportion of colorectal cancer surgeries was more variable during the COVID period (Figure 1). The number of confirmed COVID cases in South Korea was especially high in March, August, and September 2020. The increased number of patients resulted in a change in government regulatory guidelines in May 2020. More than 5000 infections were confirmed in August.

Patients with colorectal cancer who underwent surgical treatment between March and September 2020 (the COVID period) were compared with those who underwent surgical treatment between March and September 2018 and 2019 (the pre-COVID period). Table 1 shows the overall group characteristics and operative outcomes. The mean ages of patients during the COVID and pre-COVID periods were similar (62.6 vs. 61.7 years). Although the proportions of patients aged >70 (27.1% vs. 30.3%) and >80 (6.3% vs. 8.0%) years were lower during COVID than during the pre-COVID period, these differences were not statistically significant. There were no significant differences in sex distribution (*p* = 0.532) or tumor location (*p* = 0.111) between the two periods. The proportion of patients who received any preoperative treatment was higher during the COVID period. Among rectal cancer patients, the proportion receiving preoperative chemoradiotherapy (PCRT) was 38.3% and 34.9% in COVID and pre-COVID periods, respectively. Adjuvant radiotherapy was used on 24% and 22.8% of rectal cancer patients in the COVID and pre-COVID period, respectively. The proportion of PCRT was significantly higher and the proportion of adjuvant radiotherapy was not different.

Tumor-related complications, such as perforation, abscess, or obstruction, were similar between the two periods.

The number of patients who did not undergo tumor resection was significantly higher during the COVID period (*p* < 0.001). Moreover, a higher proportion of patients received palliative therapy, such as stomy formation or bypass operation, during COVID than during the pre-COVID period (4.1% vs. 1.8%).

### 3.2. Surgical Treatment and Tumor Aggressiveness in Patients Who Underwent Radical Resection

A significantly lower percentage of patients underwent minimally invasive surgery during the COVID period (81.2% vs. 88.0%, *p* < 0.001; Table 2), but there was no difference in the proportion of patients who underwent minimally invasive surgery in the absence of chemotherapy. The proportion of patients requiring resection of adjacent organs was higher during the COVID period, regardless of whether PCRT was given.

The proportion of patients who experienced tumor-related complications, such as perforation, abscess formation, and obstruction, was lower than the proportion who did not experience these complications during both the COVID and pre-COVID periods. Complication rates did not differ between patients who underwent radical resection with and without chemotherapy during both the COVID (*p* = 0.365) and pre-COVID (*p* = 0.411) periods. However, a lower percentage of tumor-related complications was observed during the COVID period in all groups. 

Higher percentages of patients in the COVID period required combined resection of organs adjacent to the tumor, both among those who did (4.8% vs. 2.8%, *p* = 0.017) and did not (5.0% vs. 2.8%, *p* = 0.017) receive chemotherapy. The adjacent organs most frequently involved during both periods were those in the GI tract (stomach and small bowel) and urinary system (bladder, ureter, and kidneys) (Table 3). Although the bladder was the most frequently involved organ during the COVID period, there were no significant relationships between organ and period. 

**Figure 2:**
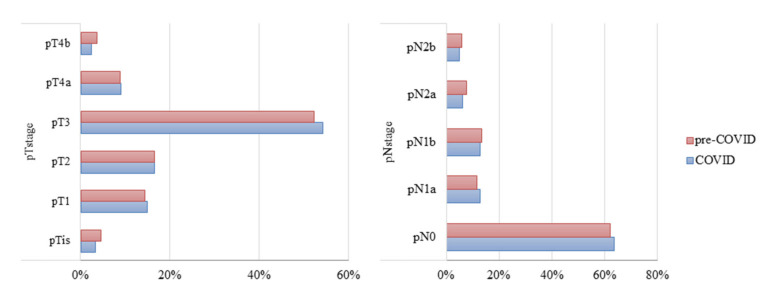
Pathologic TN stages of patients with colorectal cancer who did and did not receive chemotherapy during the pre-COVID and COVID periods.

The pathological TNM stage distribution did not differ significantly between the pre-COVID and COVID periods (Figure 2). The proportions of patients with tumors limited to the bowel (Tis to T2) and outside the bowel wall (T3 to T4) were 34.5% and 65.4% during the pre-COVID period and 35.4% and 64.6% during the COVID period. Moreover, distributions of pathologic stage did not differ in patients who did not receive chemotherapy during the two periods.

Evaluation of N stage classification showed that a higher proportion of patients had 0–1 lymph node metastases during the pre-COVID period, whereas a high proportion had greater than N1b stage during the COVID period. The proportion with over two lymph node metastases was higher during the COVID period, both among patients who did (26.4% vs. 23.7%) and did not (26.6% vs. 24.8%) receive chemotherapy. However, overall TN stages were similar during both periods. 

The incidence of lymphovascular invasion among patients who underwent radical resection for stage I to III without preoperative chemoradiotherapy was significantly higher during the COVID period (45.2% vs. 37.3%, *p* = 0.001). By contrast, the proportion with perineural invasion did not differ significantly during the two periods (24.4% vs. 22.6%, *p* = 0.366).

## 4. Discussion

This study assessed the influence of COVID-19 on surgical treatment for colorectal cancer. We found that the distribution of pathologic stages did not differ during the COVID and pre-COVID periods. The use of minimally invasive surgery, including laparoscopic and robotic surgery, was significantly lower during the COVID period. Moreover, the percentage requiring resection of adjacent organs due to tumor involvement was higher during the COVID period. These findings suggest that COVID may have influenced the medical care of patients with colorectal cancer. 

The demographic and clinical characteristics of the included patients were typical of those observed in South Korean colorectal cancer patients treated at our institution. Our hospital is a tertiary specialized medical facility, also called an advanced cancer treatment center. Multidisciplinary treatment teams are involved in patient assessment, evaluation, examination, and treatment, assessing over 3000 colorectal cancer patients annually. The present study was designed to evaluate how COVID affected colorectal cancer patient distributions and treatment approaches. 

The COVID pandemic is responsible for health emergencies worldwide. As the number of confirmed patients doubled, regulatory guidelines became stricter than during the early stages of COVID [3]. The WHO has emphasized the importance of individual hygiene [1]. This guideline is also likely to influence cancer treatments and outcomes. Cancer diagnosis rates have decreased during the pandemic because people are hesitant to visit hospitals, which are associated with high risks of infection [9,12]. Outpatient visits during the COVID-19 pandemic were found to decrease across all specialties and age groups, including cancer screening [13,14,15]. However, our cohort study showed that the gaps between the two periods in the number of total patients and surgical procedures were not meaningful. Lower diagnosis rates result mainly from primary and secondary hospitals, where they perform health screening examinations, rather than from tertiary medical centers. 

The proportion of elderly individuals was markedly lower during the COVID period. Social restrictions were emphasized for the most vulnerable age groups, not only to maintain individual health but also to prevent the spread of disease [16]. Except for esophageal cancer, patients with all other types of cancer, including breast (63.0 ± 13.0 vs. 64.3 ± 12.7 years, *p* < 0.001) and colorectal (65.4 ± 13.3 vs. 66.7 ± 13.4 years, *p* < 0.001) cancer, were found to be younger during COVID than during the pre-COVID period [17]. The lower proportion of elderly individuals was likely due to strict guidelines to remain at home and anxiety about infectious diseases. 

Because COVID spread worldwide within 1 year, the effects of COVID on cancer is likely dependent on the type and stage of cancer. The National Health Insurance Service in South Korea reported that fewer patients were diagnosed with the top five cancers (stomach, colon, cervix, liver, and breast) after COVID [18]. Concerns have arisen about missing treatments during the COVID pandemic, which may increase the mortality rate among cancer patients. However, contradictory results regarding the influence of COVID on treatment outcomes in cancer patients were observed. COVID-associated delays would likely not be fatal in patients with breast and prostate cancer, especially during their early stages [19,20]. By contrast, the proportion of lung cancer patients diagnosed with advanced non-small-cell lung cancer was higher during the COVID period [4].

At an advanced cancer treatment institution in the U.K., complete resection of colorectal tumors was performed without difficulty and without an increased risk of COVID [21]. Delays within 1 month due to COVID were acceptable and did not affect the mortality rate [22,23]. However, longer delays in surgical procedures resulted in poor outcomes. For example, a retrospective study found that delaying colorectal cancer treatment for over 1 month increased patient mortality rate [7]. Delays in diagnosis and treatment during the COVID pandemic were associated with more advanced stages and poorer clinical outcomes, including higher death rates [17]. The proportion of colorectal cancer patients in Brazil presenting at an advanced clinical stage (cT4, cN+, or M1) was significantly higher during COVID than during the pre-COVID period (64% vs. 38.7%, *p* = 0.002) [24]. A higher proportion of patients requiring resection of adjacent organs indicated that the COVID pandemic affected outcomes in these patients. The higher percentages of combined resection of adjacent organs were due to bulky tumors and accompanied tumor complications, although the oncological difference was not distinct.

The effects of COVID were also shown by the reduced performance rate of minimally invasive surgery, which was associated with a higher risk of viral diffusion [25]. Surgical practices were revised to reduce exposure to any aerosols that could be carriers of infectious agents [26]. In some patients, however, the avoidance of minimally invasive surgery was not due only to separation and self-protection from the coronavirus. The cancer patients included in the present study had been screened and confirmed as being uninfected. Thus, the surgical approach was not chosen to avoid or reduce the risk of COVID infection. Rather, open surgery was regarded as appropriate for colorectal cancer based on its aggressiveness and resultant technical challenge [27]. For patients with the following circumstances, MIS was not generally recommended: any metastatic lesions or involved adjacent organs requiring resection that were not accessible with MIS, predicted severe adhesion in patients with extensive previous abdominal operation histories, and bulky tumors in which manual control was inevitable for resection or dissection. The clinical progression of colorectal cancer led to lower rates of minimally invasive surgery. 

Although cancer was thought to be exacerbated by the epidemic, more aggressive disease does not indicate a more advanced pathologic stage. Neither COVID nor colorectal cancer stage had a significant effect on pathologic tumor distribution [21,28], nor on pathologic stage in other tumor types [19,20]. The pandemic caused delays in diagnosis or treatment at any disease control stage, and this is still ongoing. Further monitoring should continue for the whole COVID period. Tumor-related complications, such as perforation, abscess, and obstruction, likely also represent aspects of cancer aggravation, but we observed no significant relationships between complication rates and COVID. Tumor-related complications are difficult to evaluate because these categories are life-threatening and few such patients were observed.

Morbidity and mortality outcomes are difficult to compare during the pre-COVID and COVID periods and reports are controversial [7,29]. Not only was each cohort confined to a different study period, but all of these study periods were under 1 year. The recent emergence of COVID-19 as an epidemic disease limits long-term analysis. In addition, this study was based on data from a single institution, which may not be representative of the national patient population. Patients who require a higher-grade procedure or treatment visit high-volume hospitals, such as tertiary medical facilities. Reluctance to visit hospitals during the pandemic period therefore did not affect the number of patients in our institution, making it difficult to determine the effect of COVID-19 on primary healthcare for colorectal cancer. When patients visit the outpatient department of a tertiary medical center, most were referred from primary and secondary hospitals. Due to the emphasis on keeping social distance during COVID, primary and secondary hospitals, which perform endoscopy actively for health screening, had lower visit rates which would be more sensitive than in a tertiary medical center [30]. 

Despite these limitations, the present study found that COVID had an impact on the severity of colorectal cancer. Follow-up is required to assess the effects of COVID on outcomes in cancer patients who were diagnosed or treated during this period.

## 5. Conclusions

Pathologic stage and short-term surgical outcomes in patients with colorectal cancer did not differ during the COVID and pre-COVID periods in our tertiary medical facility. However, surgical aggressiveness, as shown by minimally invasive surgery and resection of adjacent organs, was significantly influenced by the COVID period. In addition, resectability decreased during the COVID period. These characteristics may influence long-term oncological outcomes, indicating the need for long-term monitoring of this patient cohort. The risks associated with COVID-19 will persist for some time.

The emphasis on social distancing, which led to a reluctance to visit hospitals because of fear and worry of COVID, resulted in delayed diagnosis and treatment of malignant diseases, including colorectal cancer. These delays inevitably resulted in postponed or rescheduled clinical procedures [31,32]. This made the extent of surgical interventions wider and increased postoperative complications. A designated screening center should not be concentrated in one area to avoid infection spread and we carefully consider applying a PCR examination algorithm before clinical procedures [33]. 

The long-term impact of treatment delay in the COVID-19 pandemic period on oncologic outcomes needs to be evaluated.

## Figures and Tables

**Table 1:** Demographic and clinical characteristics and operative outcomes in colorectal cancer patients treated during the pre-COVID and COVID periods.

Patients Characteristics	Pre-COVID	COVID	*p*-Value
	(n = 1985)	(n = 916)	
Age, mean ± SD, years	62.6 ± 12.2	61.7 ± 12.1	0.065
Age groups (<70 vs. ≥70 years)			0.079
<70 years	1384 (69.7)	668 (72.9)	
≥70 years	601 (30.3)	241 (27.1)	
Age groups (<80 vs. ≥80 years)			0.128
<80 years	1827 (92)	858 (93.7)	
≥80 years	158 (8)	58 (6.3)	
Sex			0.532
Male	1160 (58.4)	524 (57.2)	
Female	825 (41.6)	392 (42.8)	
Location of tumor			0.111
Colon	1199 (60.4)	518 (56.6)	
Rectum	773 (38.9)	389 (42.5)	
Both	13 (0.7)	9 (1)	
Preoperative treatment			0.039
None	1693 (85.3)	763 (83.3)	
PCRT *	270 (34.9)	149 (38.3)	
Chemotherapy	22 (1.1)	4 (0.4)	
Tumor-related complications, Perforation/abscess/obstruction	141 (7.1)	62 (6.8)	0.814
Resection			<0.001
Resection or excision	1949 (98.2)	878 (95.9)	
Stomy or bypass	36 (1.8)	38 (4.1)	

Results reported as n (%) or mean ± standard deviation (SD); PCRT, preoperative chemoradiotherapy; * among patients with rectal cancer.

**Table 2:** Characteristics of patients who underwent curative radical resection for stage 1–3 colorectal cancer during the pre-COVID and COVID periods.

Include Patients who Receive PCRT	Pre-COVID	COVID	*p*-Value
	(n = 1662)	(n = 772)	
Age groups (<80 vs. ≥80 years)			0.15
<80 years	1519 (91.4)	719 (93.1)	
≥80 years	143 (8.6)	53 (6.9)	
Sex			0.454
Male	972 (58.5)	439 (56.9)	
Female	690 (41.5)	333 (43.1)	
PCRT (for rectal cancer alone)	224/629 (35.6)	112/304 (36.8)	0.714
Approach			<.001
Open	198 (12)	145 (18.8)	
Minimally invasive	1449 (88)	627 (81.2)	
Tumor-related complications			0.365
Perforation/Abscess/Obstruction	86 (5.2)	33 (4.3)	
Resection of adjacent organs	47 (2.8)	37 (4.8)	0.017
Brief stage			0.247
(y)p Stage 0	51 (3.1)	33 (4.3)	
(y)p Stage I	417 (25.1)	206 (26.7)	
(y)p Stage II	568 (34.2)	241 (31.2)	
(y)p Stage III	626 (37.7)	292 (37.8)	
**Include Patients Both Who Did Not** **Receive PCRT and Who Received**	**Pre-COVID**	**COVID**	***p*-Value**
	**(n = 1438)**	**(n = 660)**	
Approach			0.001
Open	114 (8.0)	93 (14.1)	
Minimally invasive	1314 (92.0)	566 (85.8)	
Tumor-related complications			0.411
Perforation/Abscess/Obstruction	83 (5.8)	32 (4.8)	
Resection of adjacent organs	40 (2.8)	33 (5.0)	0.001
Brief stage			0.345
p Stage 0	25 (1.7)	17 (2.6)	
p Stage I	365 (25.4)	180 (27.3)	
p Stage II	492 (34.2)	207 (31.4)	
p Stage III	556 (38.7)	256 (38.8)	
Lymphovascular invasion	537 (37.3)	298 (45.2)	0.001
Perineural invasion	325 (22.6)	161 (24.4)	0.366

Results reported as n (%); PCRT, preoperative chemoradiotherapy.

**Table 3:** Organs involved in patients who underwent resection of adjacent organs.

Involved Adjacent Organs	Pre-COVID(n = 47)	COVID (n = 37)
Hepatobiliary-Pancreas ^1^	1	3
GI tract ^2^	21	8
Urinary tract ^3^	13	13
Obstetric organs ^4^	6	7
Others ^5^	6	6

^1^ Liver and pancreas; ^2^ stomach, small bowel, and colon; ^3^ bladder, ureter, and kidneys; ^4^ ovary, uterus, vagina, and mesosalphix; ^5^ omentum, pelvic wall, and peritoneum.

## Data Availability

The data presented in this study are available on request from the corresponding author. The data are not publicly available due to institutional policy.
